# A study of risk factors associated with the presence of oral potentially malignant disorders: a community-based study from Northeastern Thailand

**DOI:** 10.1186/s12903-024-04554-6

**Published:** 2024-08-11

**Authors:** Pim Chiewwit, Siribang-on Piboonniyom Khovidhunkit, Chanita Tantipoj, Prangtip Worakhajit, Boworn Klongnoi

**Affiliations:** 1 Department of Oral and Maxillofacial Surgery, Faculty of Dentistry, Mahidol University, Bangkok, Thailand; 2 Department of Advance General Dentistry, Faculty of Dentistry, Mahidol University, Bangkok, Thailand

**Keywords:** Oral potentially malignant disorders, Risk factor, Thai, Oral cancer

## Abstract

**Background:**

The principal objective of this study is to ascertain the connections between well-known risk factors of oral cancer, including smoking (cigarette and tobacco), alcohol consumption, betel quid chewing, irritations in the oral cavity, history of head and neck cancer, and history of working outdoor more than 4 days/week, and the presence of OPMDs within the Thai population.

**Method:**

349,318 subjects were recruited for initial screening, then 1,483 subjects who had at least 1 risk factor and a suspicious lesion underwent comprehensive oral examinations followed by a clinical diagnosis and then received initial treatment from either oral surgeons or oral medicine specialists. Among these subjects, individuals with at least 1 risk factor and with a clinical diagnosis of OPMDs were classified as cases, while those with at least 1 risk factor but without OPMDs were categorized as controls. The case group comprised a total of 487 subjects, whereas the control group consisted of 996 subjects. Exclusion criteria were known cases of currently having oral cancer or OPMDs.

**Results:**

The outcomes of the multivariate analysis revealed that among the variables assessed, betel quid (adjusted OR 5.12 [3.93–6.68], *p* < 0.001) and smoking (adjusted OR 1.46 [1.08–1.97], *p* = 0.013), there were an association with the presence of OPMDs. Conversely, alcohol drinking, having irritations in the oral cavity, a history of head and neck cancer, and a history of working outdoors more than 4 days/week were not associated with the presence of OPMDs. Furthermore, we also study the synergistic effect of alcohol drinking, irritations in the oral cavity, history of head and neck cancer, and history of working outdoors more than 4 days/week using subgroup analysis. The analysis showed that alcohol consumption combined with smoking or betel quid chewing expressed a significantly increased risk of OPMDs, from 1.46 to 2.03 (OR 2.03 [1.16–3.56], *p* = 0.014) and from 5.12 to 7.20 (OR 7.20 [3.96–13.09], *p* < 0.001).

**Conclusion:**

Smoking and exposure to betel quid were a significant risk factors for the presence of OPMDs. The combination of alcohol with smoking or betel quid chewing was also found to increase the risk of OPMDs in this Thai northeastern population.

## Background

In 2020, the International Agency for Research on Cancer, WHO, estimated that the number of new cancer cases was 190,636 in Thailand, and lip and oral cavity cancer was the 9th most common cancer [1]. Various forms of cancer manifest within the oral and maxillofacial region [2, 3]. Predominantly, oral squamous cell carcinoma [4, 5] emerges as the prevailing malignancy, accounting for more than 90% of the neoplastic presence in this anatomical region [6]. Despite advancements in treatment modalities, the 5-year survival rate of oral cancer remains relatively low in most countries due to the inability of early detection. Early detection of malignancy can significantly decrease mortality and morbidity [7]. When it comes to screening for oral cancer, using risk-based modeling to focus on “at-risk” groups seems like a more efficient approach than the standard method of screening the entire population [8, 9]. Given the established understanding that many risk factors have been associated with OSCC, most of them shared with oral potentially malignant disorders (OPMDs) [10], this study has selected six specific risk factors based on the criteria used in prior Thai oral cancer screening [11–18]. A study in 2016 in the northeastern part of Thailand revealed that the odds of a betel nut chewer or smoker developing an OPMDs were almost eight to nine times higher than for those who did not [15]. This is in agreement with another study, also conducted in Thailand, which found that betel quid chewing and alcohol consumption increase the risk of OPMDs 4.65 and 3.40 times, respectively [18]. Some literature suggested that irritation in the oral cavity such as an improper denture, history of head and neck cancer, and prolonged exposure to sunlight were the possible risks of OPMDs [19–28]. Regarding irritation or trauma, the latest WHO consensus on OPMDs proposes that these factors should not be regarded as direct contributors to OPMDs. Instead, they are considered variables that require control prior to establishing a definitive diagnosis of OPMDs [29]. Furthermore, prior research conducted in Thailand has documented the presence of a synergistic effect when cigarette, tobacco, alcohol consumption, and betel quid chewing were combined [18].

This research analyzed six risk factors in a community-based study in northeastern Thailand. The primary objective of this research is to differentiate between those factors that hold substantial relevance to OPMDs and those that may be insignificant.

## Materials and methods

This study is conducted as a cross-sectional study and is a part of the project entitled “Development of Disease Management Model for Oral Cancer with an Integration Network of Screening, Surveillance, and Treatment in Northeast Health District” conducted for screening of oral cancer and OPMDs in the northeastern part of Thailand [30, 31].

This study was approved by the Ethics Committee approved the study of the Faculty of Dentistry/Faculty of Pharmacy, Mahidol University, and Institutional Review Board (Approval code COA.No.MU-DT-PY-IRB 2022-013.2503 and date of approval 25 March 2022). This study was in full compliance with the International Guideline for Human Research Protection including the Helsinki Declaration, the Belmont Report, CIOMS Guideline, and the International Conference on Harmonization in Good Clinical Practice. Informed consent was obtained from all subjects involved in the study.

### Population

A total of 349,318 subjects, who lived in the provinces of Buriram, Chaiyaphum, Nakhon Ratchasima, and Surin, were questioned about all 6 risk factors using a standardized objective questionnaire along with gender, age, marital status, and occupation. Studied factors including smoking (cigarette and tobacco), alcohol consumption, betel quid chewing, irritations in the oral cavity (sharp tooth, dental caries, dental calculus, periodontal disease, or ill-fitting removable denture), history of head and neck cancer, and history of working outdoor more than 4 days/week. Due to the fact that most subjects have a history of regular sunlight exposure as part of their daily activities, we have established “working outdoors more than four days per week” as a cut-off point. This determination is based on the hypothesis that the majority of individuals generally work five or more days per week.

### Inclusion and exclusion criteria

- Inclusion criteria were all individuals aged ≥ 40 years old who lived in the provinces of Buriram, Chaiyaphum, Nakhon Ratchasima, and Surin and have oral lesions with at least 1 risk factor.
- Exclusion criteria were known cases of currently having oral cancer or OPMDs.

### Data collection

- Subjects with at least 1 factor were referred for visually screened intra-orally for OPMDs or any oral lesions by dental hygienists or local dentists, who had received prior formal training, at a subdistrict hospital. A clinical diagnosis was given as OPMDs, not OPMDs, or suspected OPMDs but not sure. Subjects with lesions were referred to specialists in oral medicine or oral and maxillofacial surgeons for further examination.
- Subjects with at least 1 factor along with suspicious lesions were re-examined by specialists in oral medicine or oral and maxillofacial surgeons from the Faculty of Dentistry, Mahidol University, or Mahurat Nakhon Ratchasima Hospital. All specialists who participated in this study had undergone discussion for proper examination and diagnosis. At the district hospital, the examination of the oral cavity was conducted under ample light illumination from a dental unit, followed by digital palpation of the lesions. Detailed information regarding the lesions, including site, size, and characteristics, is recorded. A clinical diagnosis is made based on the definition of Oral Potentially Malignant Disorders (OPMDs) provided by Warnakulasuriya S., et al. in 2020 [29]. These disorders include oral leukoplakia, oral erythroplakia, oral submucosal fibrosis, oral lichen planus/oral lichenoid lesions, oral lupus erythematosus, actinic cheilitis, and other OPMDs. After clinical diagnosis was made, subjects received initial treatments such as medication or tissue biopsy, followed by appropriate follow-up.

### Sample size calculation

The sample size was calculated based on the study of the prevalence of oral premalignant lesions conducted in Thailand by Juntanong et al [15].

$${\text{n}}\,{\text{ = }}\,{{\text{Z}}^{\text{2}}}{\text{(PQ)/(dP}}{{\text{)}}^{\text{2}}}$$

- n = sample size
- Z = 1.96
- *P* = 0.038
- d = 0.06

The calculated sample size (n) is approximately 1083 subjects.

### Statistical analysis

The data were analyzed using the IBM SPSS Statistics for Windows, version 25.0 (IBM Corp., Armonk, NY, USA). The baseline characteristics were analyzed with descriptive statistics by frequency and percentage in categorical data, mean, standard deviation, median, and interquartile range were used in continuous data. Continuous variables were compared using the independent T-test, and categorical variables were compared using the Chi-squared test or Fisher’s exact test, as appropriate.

The risk factors associated with OPMDs were selected in the logistic regression analysis performed by univariate and multivariate analysis. The odds ratio (95% CI) was presented with a *p*-value < 0.05 and was considered statistically significant.

## Result

### Participant recruitment

Individuals with at least 1 risk factor and with a clinical diagnosis of OPMDs were classified as cases, while those with at least 1 risk factor but without lesions were categorized as controls. The case group comprised a total of 487 subjects, whereas the control group consisted of 996 subjects.

### Subject characteristics

In this study, the participant distribution showed a higher number of females (64.7%) compared to males (35.3%). Additionally, there was a discernible difference in the prevalence of risk factors between genders: smoking and alcohol consumption were predominantly observed among male participants, whereas betel quid chewing was most encountered among female participants. Regarding the association between risk factors and OPMDs cases, it’s noteworthy that within the case group, a higher proportion of females (73.5%) were observed compared to males (26.5%). Furthermore, our findings indicate that within the case group, 29.2% are smokers, 26.7% had a history of alcohol consumption, 60% were betel nut chewers, 93.2% had oral cavity irritations, 14.2% had a previous head and neck cancer diagnosis, and 46% were outdoor workers (Table 1).

**Table 1:** Demographic data of the participants (*N* = 1483)

Data	Total (*n* = 1483)	Case (*n* = 487)	Non-case (*n* = 996)	*p*-value
Age (year), mean ± S.D.	67.13 ± 10.16	68.69 ± 9.71	66.37 ± 10.29	< 0.001*
Sex, %				
Male	524 (35.3)	129 (26.5)	395 (39.7)	< 0.001*
Female	959 (64.7)	358 (73.5)	601 (60.3)	
Smoking, %				
Yes	425 (28.7)	142 (29.2)	283 (28.4)	0.766
No	1058 (71.3)	345 (70.8)	713 (71.6)	
Alcohol drinking, %				
Yes	397 (26.8)	130 (26.7)	267 (26.8)	0.963
No	1086 (73.2)	357 (73.3)	729 (73.2)	
Chew betel nuts, %				
Yes	521 (35.1)	292 (60.0)	229 (23.0)	< 0.001*
No	962 (64.9)	195 (40.0)	767 (77.0)	
Irritation in mouth, %				
Yes	1390 (93.7)	454 (93.2)	936 (94.0)	0.575
No	93 (6.3)	33 (6.8)	60 (6.0)	
Cancer history, %				
Yes	189 (12.7)	69 (14.2)	120 (12.0)	0.250
No	1294 (87.3)	418 (85.8)	876 (88.0)	
Outdoor worker, %				
Yes	605 (40.8)	224 (46.0)	381 (38.3)	0.004*
No	878 (59.2)	263 (54.0)	615 (61.7)	

* Statistically significant at p-value < 0.05 determined by chi-square test and t-test

### Factors associated with the presence of OPMDs

A univariate analysis revealed that OPMDs were frequently observed among females (OR 1.82 [1.44–2.31.

], *p* < 0.001). Additionally, individuals who chewed betel quid (OR 5.02 [3.97–6.34], *p* < 0.001), and worked outdoors (OR 1.38 [1.10–1.71], *p* < 0.004) were more likely to have OPMDs.

In the multivariate analysis, the result showed that females have a 1.5 times greater risk of developing OPMDs compared to males (adjusted OR 1.50 [1.12–2.01], *p* = 0.007). Smoking (adjusted OR 1.46 [1.08–1.97], *p* = 0.013), and betel quid chewing (adjusted OR 5.12 [3.93–6.68], *p* < 0.001) were associated with the presence of OPMDs. Conversely, factors such as oral cavity irritation, a history of head and neck cancer, and outdoor work were not found to be related to the presence of OPMDs. The univariate and multivariate analyses assessing the association between risk factors and the presence of OPMDs are presented in Table 2.

**Table 2:** Univariate and multivariate analyses of risk factor of the oral potentially malignant disorders (*N* = 1,483)

Factor	Univariable analysis			Multivariable analysis		
	Crude OR	95%CI	*p*-value	Adjusted OR	95%CI	*p*-value
Age (year), mean ± S.D.	1.02	1.01–1.03	< 0.001*	0.99	0.98–1.01	0.299
Sex						
Male	Ref.			Ref.		
Female	1.82	1.44–2.31	< 0.001*	1.50	1.12–2.01	0.007*
Smoking						
Yes	1.04	0.82–1.32	0.766	1.46	1.08–1.97	0.013*
No	Ref.			Ref.		
Alcohol drinking						
Yes	0.99	0.78–1.27	0.963	1.30	0.97–1.74	0.081
No	Ref.			Ref.		
Chew betel nuts						
Yes	5.02	3.97–6.34	< 0.001*	5.12	3.93–6.68	< 0.001*
No	Ref.			Ref.		
Irritation in mouth						
Yes	0.88	0.57–1.37	0.575	1.02	0.64–1.65	0.921
No	Ref.			Ref.		
Cancer history						
Yes	1.21	0.88–1.66	0.251	1.25	0.88–1.76	0.207
No	Ref.			Ref.		
Outdoor worker						
Yes	1.38	1.10–1.71	0.004*	1.13	0.89–1.45	0.315
No	Ref.			Ref.		

* Statistically significant at p-value < 0.05 determined by logistic regression, Ref. = Reference

### Subgroup analysis of factors associated with the presence of OPMDs

Although prior analyses did not identify alcohol drinking, oral cavity irritation, a history of head and neck cancer, or outdoor work as risk factors for OPMDs, we conducted a subgroup analysis to explore potential synergistic effects among these factors. Other combinations of risk factors do not show any increased risk of OPMDs, as shown in Tables 3 and 4, and 5. According to Table 6, the analysis indicates that smoking (adjusted OR 1.46, as shown in Table 2), when combined with alcohol consumption, increases the risk of OPMDs to an adjusted OR of 2.03 (95% CI: 1.16–3.56, *p* = 0.014). Furthermore, if betel quid chewing (adjusted OR 5.12 as shown in Table 2) is combined with alcohol consumption, the risk increased to 7.20 (OR 7.20 [3.96–13.09], *p* < 0.001). Other combinations of risk factors did not show any increased risk of OPMDs, as shown in Tables 3 and 4, and 5.

**Table 3:** Subgroup analysis of factors associated with the presence of OPMDs (*N* = 1,483)

Factor	Irritation in mouth group					
	Yes			No		
	Adjusted OR	95%CI	*p*-value	Adjusted OR	95%CI	*p*-value
Smoking	1.36	1.00-1.86	0.051	3.98	1.04–15.23	0.043*
Drinking alcohol	1.26	0.93–1.71	0.129	1.05	0.28–3.96	0.941
Chew betel nuts	5.46	4.14–7.21	< 0.001*	2.53	0.86–7.43	0.090
Cancer history	1.28	0.89–1.84	0.181	1.17	0.29–4.65	0.828
Outdoor worker	1.06	0.82–1.37	0.660	2.86	0.98–8.31	0.054

* Statistically significant at p-value < 0.05 determined by logistic regression

**Table 4:** Subgroup analysis of factors associated with the presence of OPMDs (*N* = 1,483)

Factor	Cancer history group					
	Yes			No		
	Adjusted OR	95%CI	*p*-value	Adjusted OR	95%CI	*p*-value
Smoking	2.28	1.04–4.96	0.039*	1.39	1.00-1.93	0.052
Drinking alcohol	1.00	0.44–2.26	0.996	1.35	0.98–1.86	0.064
Chew betel nuts	3.26	1.52–6.96	0.002*	5.75	4.30–7.70	< 0.001*
Irritation in mouth	1.07	0.33–3.45	0.915	1.02	0.59–1.74	0.948
Outdoor worker	0.30	0.15–0.61	0.001*	1.42	1.09–1.85	0.010*

* Statistically significant at p-value < 0.05 determined by logistic regression

**Table 5:** Subgroup analysis of factors associated with the presence of OPMDs (*N* = 1,483)

Factor	Outdoor worker group					
	Yes			No		
	Adjusted OR	95%CI	*p*-value	Adjusted OR	95%CI	*p*-value
Age (year)	0.99	0.97–1.01	0.191	1.00	0.98–1.02	0.782
Sex: Female	2.44	1.50–3.98	< 0.001*	1.07	0.73–1.57	0.743
Smoking	1.58	0.96–2.59	0.074	1.53	1.03–2.25	0.033
Drinking alcohol	1.23	0.75-2.00	0.409	1.24	0.85–1.82	0.263
Chew betel nuts	4.23	2.81–6.35	< 0.001*	6.71	4.64–9.70	< 0.001*
Irritation in mouth	0.64	0.33–1.27	0.202	1.78	0.86–3.71	0.121
Cancer history	0.43	0.24–0.77	0.004	2.93	1.86–4.63	< 0.001*

* Statistically significant at p-value < 0.05 determined by logistic regression

**Table 6:** Subgroup analysis of factors associated with the presence of OPMDs (*N* = 1,483)

Factor	Alcohol drinking group					
	Yes			No		
	Adjusted OR	95%CI	*p*-value	Adjusted OR	95%CI	*p*-value
Smoking	2.03	1.16–3.56	0.014*	1.31	0.90–1.90	0.155
Chew betel nuts	7.20	3.96–13.09	< 0.001*	4.96	3.65–6.73	< 0.001*
Irritation in mouth	0.73	0.23–2.28	0.585	1.09	0.64–1.86	0.745
Cancer history	1.31	0.66–2.58	0.443	1.22	0.82–1.83	0.325
Outdoor worker	0.88	0.52–1.50	0.636	1.22	0.92–1.61	0.165

* Statistically significant at p-value < 0.05 determined by logistic regression

## Discussion

Multivariate analysis suggests that smoking, and betel quid chewing related to OPMDs with significant associations. The individuals who engaged in betel quid chewing were found to possess a substantially elevated risk, with a 5.12-fold increase in the likelihood of developing OPMDs in comparison to their non-betel quid-using counterparts. It is noteworthy that this risk was found to be more pronounced in the female population, where the prevalence of lesions was higher when compared to males. Our results align with those studies conducted in northeastern Thailand, which similarly identified a 4.11-fold increased risk of developing lesions among betel quid users compared to non-users [32]. The explanation for the higher prevalence of lesions among women in the context of betel quid chewing could be attributed to the nature of this behavior among elderly women in Southeast Asia. It is well-documented that betel quid chewing is a prevalent cultural practice in this region, particularly among older women [33]. Betel quid could cause malignant transformation due to its substances that cause genetic changes. In an in vitro study with fibroblasts from the oral epithelium, it was shown that the key components of Betel quid exhibited genotoxicity, cytotoxicity, and cell division properties [34–36].

Smoking, both cigarette and tobacco, was also identified as a potential risk factor for OPMDs in this study with statistical significance. It is noteworthy that these findings are in agreement with results reported in other studies [11–18, 37]. Studies conducted in India found that the high prevalence of OPMDs is most likely attributed to the widespread practice of chewing tobacco, with oral submucous fibrosis being the most prevalent [38–43].

More than 90% of patients in both groups exhibited oral cavity irritation, establishing it as markedly predominant among the six factors considered. Such prevalence can potentially generate distortions in statistical analysis, particularly when considering multi-causal models. According to multicausality theories, the frequency of an event is more closely related to variations in the frequency of less frequent causes. Given the high frequency of irritation within the included population, its impact cannot be considered a risk factor; however, the effects of less prevalent variables can be discerned. The presence of oral cavity irritation, whether stemming from dental calculus, periodontal disease, ill-fitting removable dentures, or defective teeth, was observed to be common within populations with limited access to dental services. It is possible that irritation is a basic characteristic in the population, which allows the effect of other factors. Previous studies have suggested that oral cavity irritation, when considered in conjunction with other factors, serves as a significant risk factor for OPMDs [44]. However, in our study, the majority of lesions in the oral cavity irritation group were not OPMDs or malignancies. As a result, we may infer that oral cavity irritation does not correlate with the development of OPMDs. This observation is in line with other research that has reported that the primary issue among denture wearers is often traumatic ulcers [45, 46]. Furthermore, other forms of mechanical trauma are generally considered reversible and are not typically associated with the development of oral potentially malignant disorders [47, 48]. Even though irritation is not a risk factor for the appearance of OPMD, it is relevant to clarify that this does not imply ruling out its possible role in carcinogenesis [49]. Taken to the clinic, this implies that irritation may not be the primary cause of oral cancer precursor lesions but could potentially act as an aggravating factor.

Our study did not find a statistically significant association between a history of head and neck cancer and the presence of OPMDs. The finding aligns with the report by Mehdi et al [50], which documented a relatively low incidence of second primary cancers at 1.14%. One plausible explanation for this discrepancy may be attributed to the heightened awareness observed among individuals in our population who had a history of head and neck cancer. It is conceivable that these individuals were more committed to regular check-up routines, thus facilitating early detection and management, potentially reducing the risk of OPMD development. This observation aligns with the findings of Bhat et al., 2019 [51], who reported a strong association between periodontitis and OPMDs after controlling for confounders. Nevertheless, a preliminary finding on the relationship between OPMDs and patterns of second primary oral cancer in betel-nut chewers indicates a strong and positive association, particularly among patients with multiple potential carcinogenic personal habits [28].

Prolonged exposure to sunlight has been identified as a cause of actinic keratosis, which is one of the OPMDs [29]. Also both UVB and UVA radiation has been widely acknowledged to potentially lead to DNA mutations, with subsequent cancer development [52, 53],40] 41]. These malignancies tend to manifest in areas of the body that are frequently exposed to sunlight, such as the face and lips. However, the findings of our study have revealed no apparent correlation between such radiation exposure and OPMDs development. This discrepancy in outcomes may be attributed to the naturally darker skin tone prevalent within our study population. It is well-documented that darker skin tones benefit from inherent protections afforded by heightened epidermal melanin content, increased melanocyte activity, and larger and more dispersed melanosomes. These factors collectively enable them to effectively filter and block a significantly greater portion of ultraviolet B (UVB) radiation compared to lighter skin tones [54]. Moreover, outdoor workers in Thailand often adopt protective measures by wearing appropriate gear that covers their faces. This conscientious practice serves to diminish the likelihood of sunlight exposure, further contributing to the observed lack of correlation between sun exposure and OPMDs development.

With regard to synergistic effects involving multiple risk factors, subgroup analysis shows that alcohol consumption combined with smoking or betel quid chewing expresses a significantly increased risk of OPMDs. This observation aligns with findings from other studies, such as prior research conducted in Thailand has documented the presence of a synergistic effect when cigarette smoking, tobacco use, alcohol consumption, and betel quid chewing were combined [18]. Similarly, a study conducted in Sri Lanka has provided evidence that the combination of betel quid and tobacco usage significantly amplifies the risk of developing OPMDs, with the risk escalating from 5.5 to 14.9-fold [55]. Tables 6 and 4, and 5 showed a significantly higher risk for individuals who chew betel nut, with or without another factor. This may be attributed to the significant influence of betel nut chewing, which could confound the results of subgroup analyses. However, we did not observe any synergistic effects among irritation in the oral cavity, a history of head and neck cancer, and outdoor work.

## Conclusion

This study’s findings indicated that smoking and exposure to betel quid were a significant risk factors for the presence of OPMDs. Moreover, a combination of alcohol with smoking or betel quid chewing was also found to increase the risk of OPMDs in our population.

On the other hand, alcohol consumption, irritation in the oral cavity, history of head and neck cancer, and working outdoors more than 4 days/week were not found to be risk factors in northeastern Thai people. Additionally, subgroup analysis of irritation in the oral cavity, history of head and neck cancer, and outdoor work more than four days per week reveals no increased risk of OPMDs.

## Limitation

In this study, the method of classification into the case group relies on clinical diagnosis, prompting concerns about the accuracy of diagnosis. Multiple previous studies have reported compatibility rates of 50-81.2% for clinical and histopathological diagnosis [56–59]. Nevertheless, study developed in the United States, 976 patients were evaluated, and both the presumptive clinical diagnosis and the final histopathological diagnosis for each subject were recorded. The outcomes revealed that the rate of wrong diagnosis for cancerous lesions was only 5.6% and the highest rate of wrong diagnosis was related to benign lesions such as hyperkeratosis and, fibroma [56].

Despite the use of a validated questionnaire for recording variables, the questionnaire did not categorize chronic irritation and acute irritation not associated with carcinogenesis. Therefore, there may be an overrepresentation of individuals in the irritation group. Regarding the validity of using patients’ self-reported perception of irritation to diagnose injury associated with irritation, there may be an over- or under-reporting of individuals in this group. This issue has been examined in only one study, which demonstrates that self-perception does not align with the diagnosis of irritation lesions [60].

## Data Availability

The datasets used and/or analyzed during the current study are not publicly available due to the confidentiality of the participants but are available from the corresponding author on reasonable request.

## Abbreviations

OSCC: Oral squamous cell carcinoma
OPMDs: Oral potentially malignant disorders
CI: Confidence interval
OR: Odds ratio
SD: Standard deviation
WHO: World Health Organization
